# Potential biomarkers of Alzheimer’s disease and cerebral small vessel disease

**DOI:** 10.3389/fnmol.2022.996107

**Published:** 2022-10-10

**Authors:** Chun Zou, Xiaohua Huang, Yilong Zhang, Mika Pan, Jieqiong Xie, Liechun Chen, Youshi Meng, Donghua Zou, Jiefeng Luo

**Affiliations:** ^1^Department of Neurology, The Second Affiliated Hospital of Guangxi Medical University, Nanning, China; ^2^Department of Neurology, The Affiliated Hospital of Youjiang Medical University for Nationalities, Baise, China; ^3^Clinical Research Center, The Second Affiliated Hospital of Guangxi Medical University, Nanning, China; ^4^Department of Neurology, The Fifth Affiliated Hospital of Guangxi Medical University, Nanning, China

**Keywords:** Alzheimer’s disease, cerebral small vessel disease, bioinformatics, SIRT1, metabolic pathways

## Abstract

**Background:**

Cerebral small vessel disease (CSVD) is associated with the pathogenesis of Alzheimer’s disease (AD). Effective treatments to alleviate AD are still not currently available. Hence, we explored markers and underlying molecular mechanisms associated with AD by utilizing gene expression profiles of AD and CSVD patients from public databases, providing more options for early diagnosis and its treatment.

**Methods:**

Gene expression profiles were collected from GSE63060 (for AD) and GSE162790 (for CSVD). Differential analysis was performed between AD and mild cognitive impairment (MCI) or CSVD progression and CSVD no-progression. In both datasets, differentially expressed genes (DEGs) with the same expression direction were identified as common DEGs. Then protein-protein interaction (PPI) network was constructed for common DEGs. Differential immune cells and checkpoints were calculated between AD and MCI.

**Results:**

A total of 146 common DEGs were identified. Common DEGs were mainly enriched in endocytosis and oxytocin signaling pathways. Interestingly, endocytosis and metabolic pathways were shown both from MCI to AD and from CSVD no-progression to CSVD progression. Moreover, SIRT1 was identified as a key gene by ranking degree of connectivity in the PPI network. SIRT1 was associated with obesity-related genes and metabolic disorders. Additionally, SIRT1 showed correlations with CD8 T cells, NK CD56 bright cells, and checkpoints in AD.

**Conclusion:**

The study revealed that the progression of AD is associated with abnormalities in gene expression and metabolism and that the SIRT1 gene may serve as a promising therapeutic target for the treatment of AD.

## Introduction

Alzheimer’s disease (AD) is an incurable and neurodegenerative disease characterized pathologically by the presence of neurofibrillary tangles and amyloid-beta (Aβ) plaques in the brain (Jian et al., 2016, 2021). According to current estimates, 17% of the population in the USA aged 75–84 years suffer from AD, and the disease is responsible for $236 billion annually in the country dedicated to care for the AD patients. Recent data suggest that the prevalence of dementia will double in Europe and globally by 2050 (Scheltens et al., 2021; Luo et al., 2022).

In 2018, the National Institute on Aging and the Alzheimer’s Association proposed the Aβ, tau, neurodegeneration research framework for defining and diagnosing AD (Jack et al., 2018; Ma et al., 2022). Despite extensive research, the etiology of AD remains unclear. It is need to develop treatments with disease-modifying effects to halt the disease at the earliest preclinical stage of the disease to preserve the brain’s cognitive function. Mild cognitive impairment (MCI) often precedes clinical dementia, and MCI is considered an important risk factor for the future development of AD (Ritchie et al., 2014). Appropriate preventive interventions help slow the progression of MCI to dementia (Martinez et al., 2017).

Cerebral small vessel disease (CSVD) is a group of pathological processes with diverse etiologies and pathogenesis, affecting cerebral arterioles and microvessels (Jimenez-Sanchez et al., 2021). Although cerebral CSVD independently contributes to morbidity, disability, and mortality, CSVD is the most common cause of cognitive impairment and AD (Yi et al., 2018). CSVD is implicated in AD development, with CSVD augmenting or modifying the progression of AD (Cai et al., 2015; Low et al., 2021). Similar risk factors (Kim H. W. et al., 2020) and pathophysiological mechanisms (Liu et al., 2018) were shared in AD and CSVD. Moreover, it is of great interest and potential to explore the role of immune dysfunction induced by immune cells in CSVD and corresponding brain damage in AD (Jian et al., 2020). Importantly, imaging examinations reveal a direct relationship between the occurrence of AD and CSVD (Litak et al., 2020).

White matter hyperintensities (WMHs) have long been considered radiological surrogate markers for CSVD (Kalaria and Sepulveda-Falla, 2021). WMHs are also a core feature of familial AD pathology (Lee et al., 2016). Although numerous studies support the idea that treatment and prevention of CSVD will benefit AD, there is a lack of correlation studies considering longitudinal changes in CSVD with AD-related progression.

In the present study, we explored markers and underlying molecular mechanisms associated with the AD disease course by utilizing gene expression profiles of AD and CSVD patients from public databases, providing more options for diagnosis and prevention of early to late AD progression.

## Material and methods

### Data collection

Gene expression profiles of AD and CSVD were downloaded from the gene expression omnibus (GEO) database. GSE63060 (Sood et al., 2015) included gene expression profiles of blood samples from 145 AD subjects, 80 MCI subjects, and 104 healthy controls based on the GPL6947 platform. GSE162790 (Noz et al., 2021) included gene expression profiles of monocytes in blood samples from four subjects with CSVD progression and four subjects with CSVD no-progression (without incident lesions or WMH progression) based on the GPL18573 platform. Individuals with any incident lesion during the study period (diffusion-weighted imaging-positive lesion, microbleed, or lacune) and individuals belonging to the first quartile of WMH progression were classified as participants with CSVD progression.

### Differential expression analysis

Differential expression of genes between AD and MCI was analyzed using *limma* package in R (Ritchie et al., 2015). Differential expression analysis between CSVD progression and CSVD no-progression was performed using the *DESeq2* package in R (Love et al., 2014). *P* < 0.05 was used as a screening threshold to obtain differentially expressed genes (DEGs). Common DEGs were identified by intersecting the upregulated or downregulated DEGs in GSE63060 and GSE162790 datasets.

### Enrichment analysis

Gene set enrichment analysis (GSEA) for genes expressed in AD and MCI was performed using *clusterProfiler* R package (Yu et al., 2012), and results were visualized using with fgsea R package. GSEA was also used for common DEGs in AD and CSVD while SubtypeGSEA was performed to evaluate the pathways in AD, MCI, and controls. Gene Ontology (GO) and Kyoto Encyclopedia of Genes and Genomes (KEGG) pathways enrichment analyses was carried out for common DEGs with the *clusterProfiler* R package. Additionally, the activated and inhibited pathways in AD were calculated using *GSVA* R software package (Hanzelmann et al., 2013). Metascape online tool^1^ was also used to analyze the biological functions. In addition, metabolic-related signaling pathways were collected from the KEGG website^2^ for correlation analysis with pathway genes of common DEGs. *P* < 0.05 was considered as significant.

### Construction of protein-protein interaction network

According to The Search Tool for the Retrieval of Interacting Genes/Proteins (STRING) database, we obtained protein interaction data. The protein-protein interaction (PPI) network of common DEGs was constructed and displayed in Cytoscape. PPI network genes were ranked by their degree of connectivity, and the gene with a maximum degree of connectivity was identified as the key gene.

### Immune cell infiltration

CIBERSORT^3^ was used to analyze the abundance of immune cells in AD samples. The single-sample GSEA (ssGSEA) was performed using *GSVA* R software package to quantify the levels of immune cells in AD samples. Expression of immune checkpoints was detected in AD and MCI. Correlations between the key gene and immune cells or immune checkpoints were calculated using Pearson correlation analysis.

### Data analysis and statistics

In this study, all analyses were performed based on the BioInforCloud platform.^4^

## Results

### Common differentially expressed genes in Alzheimer’s disease and cerebral small vessel disease

**FIGURE 1:**
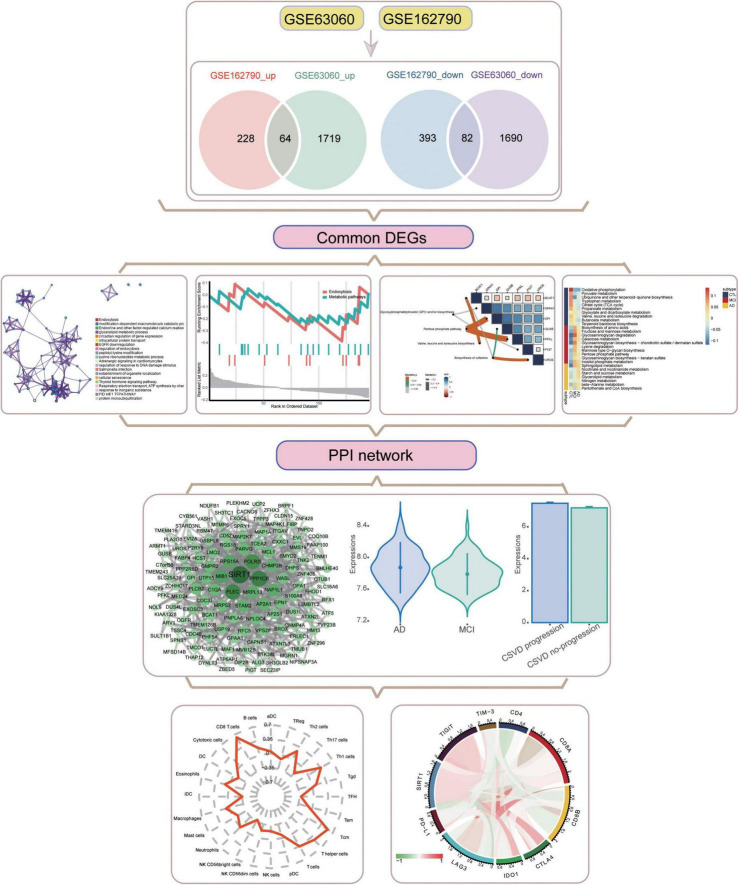
Flowchart of this study. AD, Alzheimer’s disease; CSVD, cerebral small vessel disease. DEGs, differentially expressed genes; MCI, mild cognitive impairment; PPI, protein-protein interaction.

**FIGURE 2:**
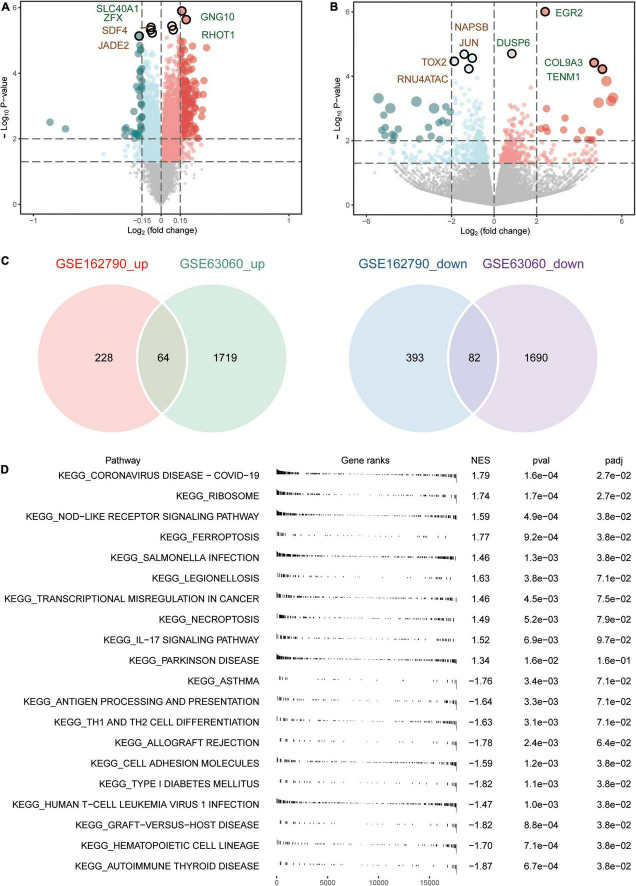
Aberrantly expressed genes in AD and CSVD progression. **(A)** Volcano plot of differentially expressed genes between AD and MCI. Red dots are up-regulated, and green dots are down-regulated. **(B)** Volcano plot of differentially expressed genes between CSVD progression and CSVD no-progression. Red dots are up-regulated, and green dots are down-regulated. **(C)** The intersection of up-regulated or down-regulated DEGs in GSE63060 and GSE162790 datasets were considered common DEGs. **(D)** KEGG pathways of GSEA in AD or MCI. NES, normalized enrichment score; *p*-val, *P*-value; *p*-adj, adjust *P*.

An overview of the study design is shown in Figure 1. A total of 3,555 DEGs between AD and MCI were identified in the GSE63060 dataset (Figure 2A), while 767 DEGs between CSVD progression and CSVD no-progression were identified in the GSE162790 dataset (Figure 2B). These DEGs were significantly different. Among these DEGs, 64 and 82 simultaneously upregulated and downregulated, respectively; hence, they were considered common DEGs (Figure 2C). Additionally, we explored the signaling pathways activated in AD or MCI patients by GSEA (Figure 2D), and we found that AD was activated in coronavirus disease, ribosome, NOD-like receptor signaling pathway, and ferroptosis. In contrast, Th1 and Th2 cell differentiation, cell adhesion molecules, and autoimmune thyroid disease were activated in MCI.

### Biological functions of common differentially expressed genes

**FIGURE 3:**
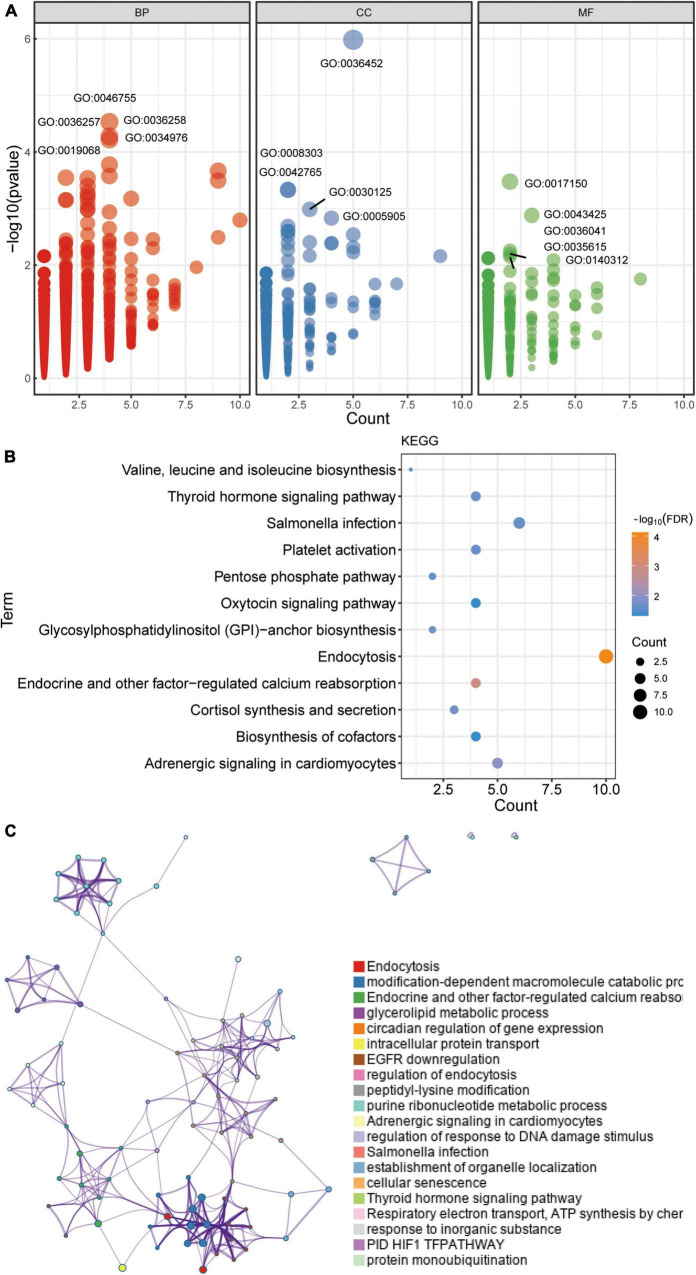
Enrichment of GO and KEGG pathways for common DEGs. **(A)** GO enrichment analysis for common DEGs, including biological processes (BP), cellular components (CC), and molecular function (MF). **(B)** KEGG pathways enrichment analysis for common DEGs. FDR, false discovery rate. **(C)** Enrichment analysis for common DEGs using Metascape online tool.

We performed an enrichment analysis to evaluate the biological function of the common DEGs. The GO results (Figure 3A) included biological processes (BPs), cellular components (CCs), and molecular functions (MFs). In BPs, GO:0034976 (response to endoplasmic reticulum stress), GO:0036258 (multivesicular body assembly), and GO:0036257 (multivesicular body organization) were found. For CCs, GO:0036452 (ESCRT complex), GO:0008303 (caspase complex), and GO:0042765 (GPI-anchor transamidase complex) were enriched. GO:0017150 (tRNA dihydrouridine synthase activity), GO:0043425 (bHLH transcription factor binding), and GO:0036041 (long-chain fatty acid-binding) were involved in MFs. Moreover, KEGG enrichment results (Figure 3B) showed that common DEGs were involved in endocytosis, oxytocin signaling pathway, and Salmonella infection. Common DEGs were also involved in endocytosis, modification-dependent macromolecule catabolic process, and endocrine and other factor-regulated calcium reabsorption in Metascape results (Figure 3C). These enrichment results were statistically significant.

**FIGURE 4:**
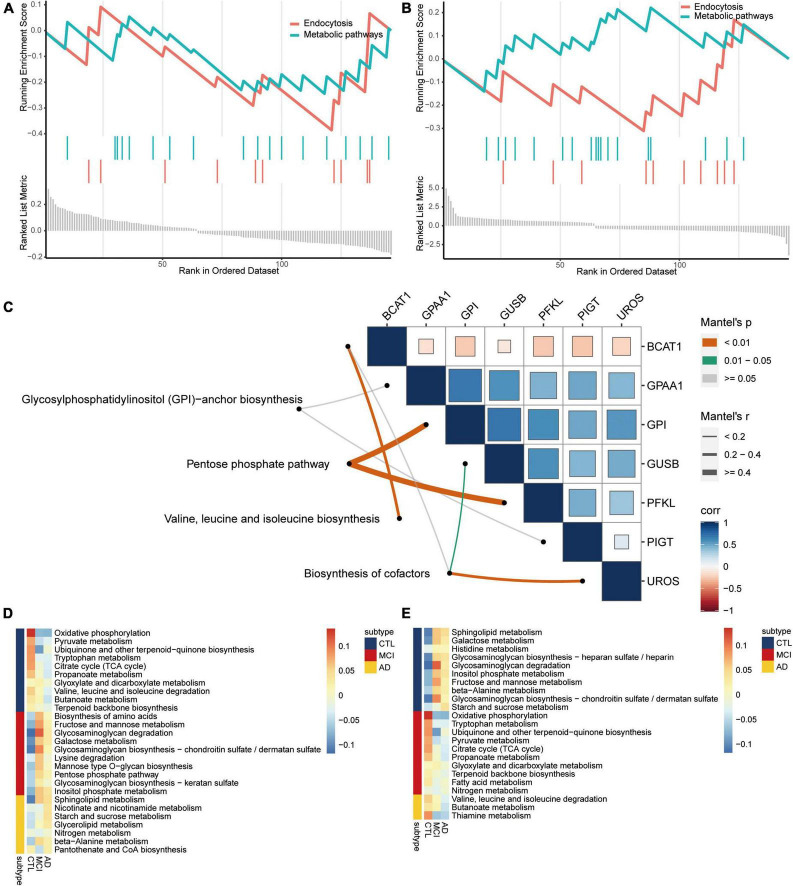
Metabolism pathways are involved in the disease course of AD. Same KEGG pathways of GSEA in AD **(A)** and CSVD **(B)**. **(C)** Correlations among metabolism pathways and common DEGs. Corr, correlation. Metabolism pathways were quantified for upregulated **(D)** or downregulated **(E)** from AD to MCI, then to controls. AD, Alzheimer’s disease; CTL, controls; MCI, mild cognitive impairment.

By screening the same KEGG pathways of GSEA in AD and CSVD, we found that endocytosis and metabolic pathways were all significantly downregulated (Figures 4A,B). By collecting metabolic-related pathways from KEGG website, we then identified four pathways [glycosylphosphatidylinositol (GPI)-anchor biosynthesis; pentose phosphate pathway; valine, leucine, and isoleucine biosynthesis; biosynthesis of cofactors] in KEGG pathways for common DEGs. The pentose phosphate pathway had the highest correlations with GPI and PFKL (Figure 4C). Moreover, we quantified the expression of metabolic pathways among AD, MCI, and controls by subtypeGSEA. Nicotinate and nicotinamide metabolism, starch and sucrose metabolism, and glycerolipid metabolism were upregulated from controls to MCI, and then to AD (Figure 4D). Valine, leucine, and isoleucine degradation, butanoate metabolism, and thiamine metabolism were downregulated from controls to MCI, and then to AD (Figure 4E). The results showed significance.

### Identification of key gene

**FIGURE 5:**
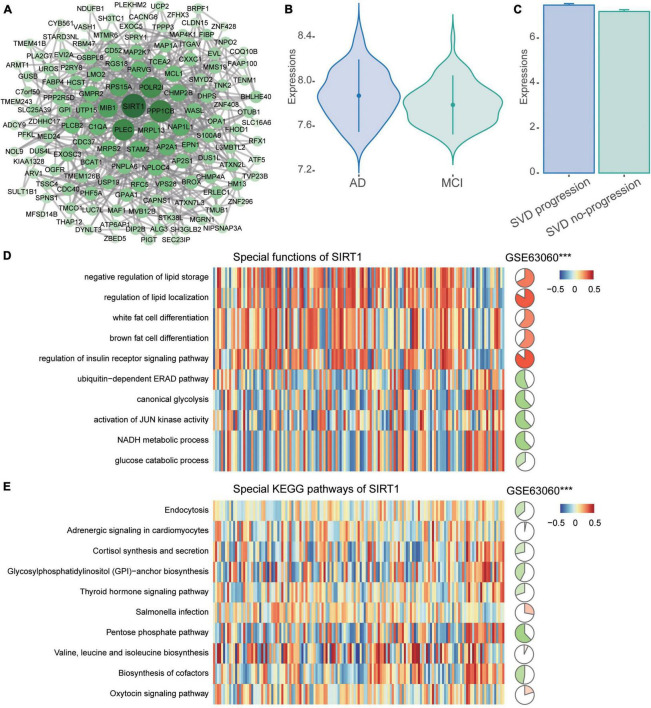
PPI network of common DEGs. **(A)** The PPI network of common DEGs was constructed using the STRING website. **(B)** Expression of SIRT1 in AD and MCI in the GSE63060 dataset. AD, Alzheimer’s disease; MCI, mild cognitive impairment. **(C)** Expression of SIRT1 in CSVD progression and CSVD no-progression in the GSE162790 dataset. CSVD, cerebral small vessel disease. **(D)** Correlation of SIRT1 with biological processes. **(E)** Correlation of SIRT1 with KEGG pathways. Red is activation, and blue is inhibition in AD in the heatmap. Red is positive correlation and green is negative correlation in circle graphs. ***P < 0.001.

The PPI network of common DEGs was then constructed. Then we identified 134 network genes (Figure 5A), and SIRT1 was identified as a key gene according to the highest degree of connectivity in the PPI network. SIRT1 was upregulated in AD compared to MCI (Figure 5B) and in CSVD progression than in CSVD no-progression (Figure 5C). In order to explore the potential biological functions of SIRT1, we calculated its correlation and BP (Figure 5D). The results showed that SIRT1 was significant more positively correlated with the regulation of insulin receptor signaling pathway, and regulation of lipid localization, while it was significant more negatively correlated with the canonical glycolysis and activation of JUN kinase activity. For the KEGG pathways (Figure 5E), SIRT1 was significant more positively correlated with valine, leucine, and isoleucine biosynthesis, and Salmonella infection, while it was significant more negatively correlated with pentose phosphate pathway and biosynthesis of cofactors.

### Immune microenvironment in Alzheimer’s disease

**FIGURE 6:**
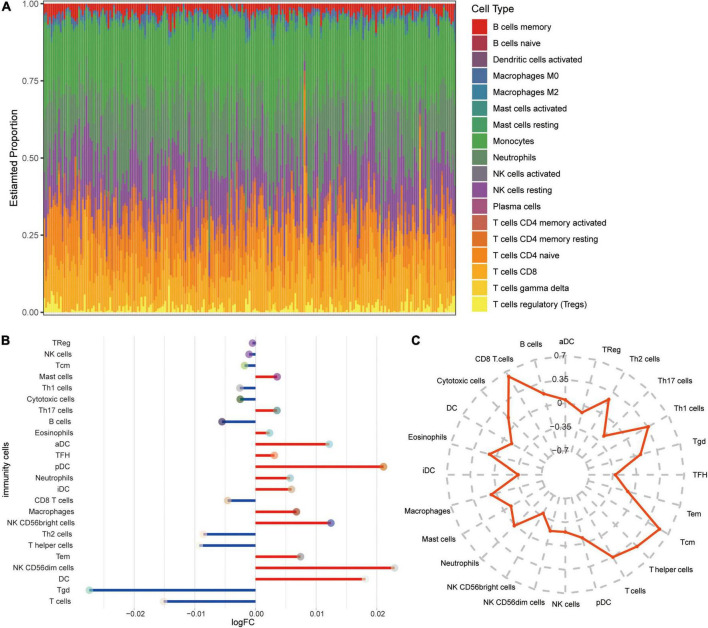
Alterations in immune cells in AD. **(A)** Estimated proportions of 24 immune cell types in AD samples of GSE63060 dataset. X-axis means AD samples. **(B)** Differential levels of immune cells between AD and MCI. Red indicates up-regulation, and green indicates down-regulation. **(C)** Correlations between immune cells and SIRT1.

To explore the altered immune microenvironment in AD patients, we first examined the abundance of immune cells using CIBERSORT (Figure 6A). The proportion of monocytes, neutrophils, resting NK cells, CD8 T cells, and T cells gamma delta was higher among all immune cells. Using ssGSEA (Figure 6B), we found significant elevated levels of neutrophils and NK CD56bright cells in AD, while T cells and CD8 T cells were significant lower in AD than in MCI. Correlations between immune cells and SIRT1 were identified (Figure 6C), and results showed that CD8 T cells and T cells had a significant high positive correlation, while NK CD56bright cells had a significant negative correlation with SIRT1.

**FIGURE 7:**
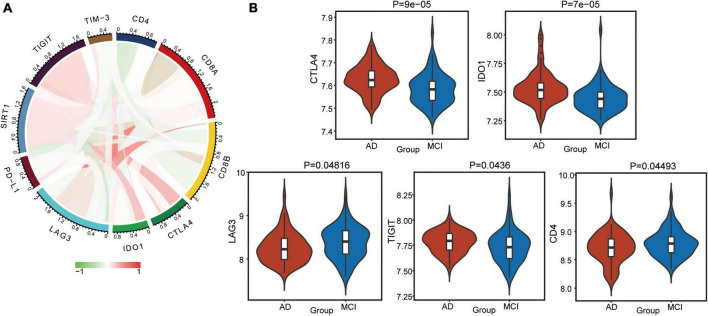
Alterations in immune checkpoints in AD. **(A)** Correlations between immune checkpoints and SIRT1. Red indicates positive correlation, and green indicates negative correlation. **(B)** Differentially expressed checkpoints between AD and MCI.

Furthermore, we calculated correlations between immune checkpoints and SIRT1 (Figure 7A). TIGIT, CTLA4, and IDO1 were significantly positively correlated with SIRT1, while CD4 and LAG3 was negatively correlated with SIRT1. The difference in checkpoints between AD and MCI was identified (Figure 7B). CTLA4, TIGIT, and IDO1 expressions were significantly higher in AD, while CD4 and LAG3 expressions were low.

## Discussion

Much of our understanding of AD pathogenesis comes from identifying key genes involved in the etiology and pathology of the disease (Zou C. et al., 2019). To date, there is no treatment to alleviate AD (Jian et al., 2017; Zou D. et al., 2019). The current study focuses on exploring the possibility of biomarkers and therapeutic targets for AD progression with reliable peripheral non-invasiveness and scalability. It is important to incorporate CSVD as a biomarker in the biological definition of AD (DeCarli et al., 2019). Probing for potential markers common to both is necessary to delay the progression of AD, although the interplay between vascular and neurodegenerative processes may be unclear. In the present study, we aimed to identify potential markers enhancing AD pathology progression by combining the disease course of CSVD and associations with the progression from MCI to AD.

We identified 146 common genes during the progression of CSVD and MCI to AD. These genes were associated with endocytosis, oxytocin signaling pathway, and calcium reabsorption; endocytosis is required for synaptic activity-dependent release of Aβ *in vivo* (Cirrito et al., 2008). Increased neuronal endocytosis in sporadic AD is a potential mechanism to promote Aβ generation and neuropathology (Cataldo et al., 1997; Zhou et al., 2021). The function of oxytocin is altered in patients with depression, AD, Parkinson’s disease, autism, and schizophrenia (Szczepanska-Sadowska et al., 2022). Increasing evidence has confirmed oxytocin’s effects on energy expenditure or metabolism (Kerem and Lawson, 2021). Oxytocin receptors (OXTR) are highly expressed in white adipose tissue and less abundant in brown adipose tissue, promoting increased energy expenditure (Yu et al., 2012; Yuan et al., 2020). Chronic oxytocin administration leads to sustained weight loss by reducing food intake, increasing energy expenditure, and inducing lipolysis (Lawson, 2017). Moreover, oxytocin exerts anti-neuroinflammatory effects by limiting oxidative stress and pro-inflammatory cascades (Friuli et al., 2021). Oxytocin stimulates arc pro-opiomelanocortin neurons by increasing calcium levels (Maejima et al., 2014).

Ca^2+^ signaling is a crucial factor in neurodevelopment, and neurodegeneration, and abnormal Ca^2+^ signaling is an important mechanism underlying synaptic weakening and elimination in AD brains (Popugaeva et al., 2017). Accumulating evidence suggests that Ca^2+^ is dysregulated in AD, which shown as apparent excess Ca2 + influx through voltage-gated Ca2 + channels, leading to the Ca^2+^ related hypothesis of brain aging and dementia (Thibault et al., 2007). Preclinical data suggest that calcium-sensing receptor inhibition may effectively counteract the pathological mechanisms of AD (Giudice et al., 2019).

The endocytosis and metabolic pathways are simultaneously present in the progression of CSVD and AD. Studies have suggested that metabolic disorders and altered levels of AD-related proteins may contribute to a higher prevalence of AD (Kim J. Y. et al., 2020). Some metabolic diseases and alterations of substances are associated with AD (Frolich et al., 1998; Kim et al., 2017). Understanding the etiology of AD has proven challenging despite a large body of literature investigating metabolic disorders and their risk factors for AD using metabolomics platforms. Generally, MCI is considered a long pre-dementia stage of AD with a cumulative progressive incidence from MCI to dementia of approximately 50% over 3 years (van Maurik et al., 2017). There are significant metabolic abnormalities in the progression of MCI to AD (Nho et al., 2021). The underlying gene and biological specificity of the MCI to AD process are pointed out, contributing to the early diagnosis and risk assessment of AD.

Our results showed that SIRT1 occupies a significant position among the common DEGs. SIRT1 can regulate many cellular cascades related to cell survival and metabolic homeostasis that may affect brain cells (Outeiro et al., 2008). SIRT1 engages in memory formation in the brain by regulating synaptic plasticity, promoting axonal extension and dendritic branching (Herskovits and Guarente, 2014). Interestingly, SIRT1 affects blood-brain barrier integrity, regulating aging-related functions in mice experiments (Stamatovic et al., 2019). There is an association between the activation of SIRT1 in the brain and the inhibition of Aβ generation (Hadar et al., 2018). SIRT1 may regulate aging and metabolic processes involved in AD pathogenesis and thus may represent a potential therapeutic target (Julien et al., 2009; Campagna et al., 2018). Unlike the above findings, our analysis revealed that SIRT1 had up-regulated expression in both AD and CSVD progression patients, which may be related to the blood sample. Kilic et al. (2015) found that SIRT1 protein expression in serum was significantly increased in elderly patients, and that there was a significant positive correlation between SIRT1 levels and age in their study population. The correlation between SIRT1 levels in peripheral blood and AD progression requires further investigation.

Additionally, SIRT1 can regulate the central nervous system’s inflammatory response, and neuroinflammation is considered the pathogenesis of AD (Jiao and Gong, 2020). The results of immune correlation analysis showed that the positive correlation between SIRT1 and CD8 T cells was high, and CD8 T cells were downregulated in AD. When SIRT1 levels are reduced, CD8 + T cell glycolysis and cytotoxicity are enhanced, resulting in immune dysfunction (Qiu et al., 2021). SIRT1 expression is significantly downregulated in terminally differentiated CD8 + memory T cells, which accumulate with age in humans (Jeng et al., 2018). In addition, the absolute number of NK cells increases with age, and CD56bright cells predominate (Kaszubowska et al., 2018). SIRT1-mediated changes in granulosome function, metabolism, oxidative stress, and cell cycle may be central to its ability to enhance immune responses (Elesela et al., 2020). However, the exact mechanism by which SIRT1 participates in AD progression needs to be further explored.

There are limitations to the present study. First, the data of our analysis were obtained from a public database, and the key results need to be thoroughly investigated utilizing in more experiments. Second, it also needs to be further explored whether an insufficient sample size and different sample types have clinical availability of key outcomes. In addition, whether the low expression of SIRT1 in peripheral blood samples is associated with AD progression needs to be further explored and validated.

## Conclusion

The present study revealed changes in genes and metabolic pathways during the progression of CSVD and AD. SIRT1 as a key gene may provide a helpful basis for the design of future AD therapeutics.

## Data availability statement

The datasets presented in this study can be found in online repositories. The names of the repository/repositories and accession number(s) can be found in the article/supplementary material.

## Ethics statement

The studies involving human participants were reviewed and approved by The Second Affiliated Hospital of Guangxi Medical University. Written informed consent for participation was not required for this study in accordance with the national legislation and the institutional requirements. Written informed consent was obtained from the individual(s) for the publication of any potentially identifiable images or data included in this article.

## Author contributions

DZ and JL conceived and designed the study. CZ, XH, and YZ collected and analyzed the data. All authors prepared the figures and tables, wrote and reviewed the manuscript, and approved this submission.

## Footnotes

1
https://metascape.org/gp/index.html#/main/step1

2
https://www.kegg.jp/

3
https://cibersort.stanford.edu/

4
http://www.bioinforcloud.org.cn
